# Deaths from Chloroform

**Published:** 1882-04

**Authors:** 


### DEATHS FROM CHLOROFORM.

Within a few weeks two patients have died in Chicago from the effects of chloroform administered for the purpose of having teeth extracted. The last case occurred December 20th. The patient was in a dentist's chair, and a physician administered the anæsthetic. Thirty minutes were required to anæsthetize the patient, and from an ounce and a half to two ounces of chloroform were administered. The patient became cyanosed and ceased to breathe after two or three teeth had been extracted. He was a man about forty years old, and was supposed to be healthy, although neither the dentist nor physician had ever seen him before or even knew his name or residence. The patient was in a sitting posture during the anæsthesia and operation.—*The Boston Med. and Surg. Journal.*

